# Telomeres and telomerase as therapeutic targets to prevent and treat age-related diseases

**DOI:** 10.12688/f1000research.7020.1

**Published:** 2016-01-20

**Authors:** Christian Bär, Maria A. Blasco

**Affiliations:** 1 Telomeres and Telomerase Group, Molecular Oncology Program, Spanish National Cancer Centre (CNIO), Madrid, Spain

**Keywords:** Telomeres, Telomerase, Aging, telomere length, short telomeres

## Abstract

Telomeres, the protective ends of linear chromosomes, shorten throughout an individual’s lifetime. Telomere shortening is a hallmark of molecular aging and is associated with premature appearance of diseases associated with aging. Here, we discuss the role of telomere shortening as a direct cause for aging and age-related diseases. In particular, we draw attention to the fact that telomere length influences longevity. Furthermore, we discuss intrinsic and environmental factors that can impact on human telomere erosion. Finally, we highlight recent advances in telomerase-based therapeutic strategies for the treatment of diseases associated with extremely short telomeres owing to mutations in telomerase, as well as age-related diseases, and ultimately aging itself.

## Telomere structure, function and maintenance

Telomeres are heterochromatic structures located at the ends of linear chromosomes formed by DNA tandem repeats bound by specialized protein complexes, which exert a protective function. A proper telomere structure prevents chromosome ends from being recognized as DNA strand breaks, thus preventing illegitimate homologous recombination between telomeres as well as chromosome end-to-end fusions ^1^. In vertebrates, telomeric DNA is composed of up to thousands of TTAGGG hexanucleotide repeats that are bound by a six-protein complex known as shelterin, which encompasses TRF1, TRF2, POT1, TIN2, TPP1, and RAP1 ^2^. TRF1 and TRF2 directly bind double-stranded telomeric repeats, whereas POT1 recognizes the single-stranded telomeric G-rich 3’ overhang. TIN2 binds to TRF1 and TRF2 through distinct domains and also recruits a TPP1-POT1 heterodimer, thus bridging different shelterins to organize the telomere cap ^2^. Finally, RAP1 is recruited to telomeres by TRF2, but can also bind throughout chromosome arms to regulate transcription, playing an important role in protection from obesity and metabolic syndrome in mice ^3– 5^. Interestingly, all shelterins except RAP1 are essential for life ^6– 8^, owing to the fact that RAP1 is the only shelterin dispensable for telomere protection ^3, 9, 10^.

Telomeres are proposed to be further stabilized by the formation of a protective T-loop lariat structure. The single-stranded 3’ overhang loops back and invades double-stranded telomeric DNA in a TRF2-dependent manner ^11, 12^. Thus, the T-loop sequesters the ends of chromosomes and provides a mechanism to prevent the full activation of a DNA damage response typically observed at most types of DNA ends ^13^.

Importantly, owing in part to the so-called “end replication problem”, telomeres shorten during each cell duplication cycle due to the inability of replicative DNA polymerases to fully replicate the 3’ ends of linear chromosomes ^14, 15^. In particular, the removal of RNA primers, which provide the required 3’OH group for addition of dNTPs by DNA polymerases, renders the newly synthesized DNA strand shorter than the parental template. Thus, chromosomes progressively shorten from both ends upon repeated cell division, a process which in the context of the organism contributes to progressive telomere shortening with aging in all cell types where it has been studied ^16^. When telomeres reach a critically short length they are detected by the DNA repair systems as DNA damage and elicit cell cycle arrest and cell death responses ^17^. Thus, telomere shortening underlies the “molecular clock” proposed by Hayflick to explain the limited lifespan of cells in culture, or “Hayflick limit” ^17, 18^.

Telomerase is a DNA reverse transcriptase polymerase (telomerase reverse transcriptase [ *TERT*]) which uses an RNA template (telomerase RNA component [ *TERC*]) for *de novo* addition of telomeric DNA onto telomeres, thus compensating for the telomere erosion caused by cell divisions ^19^. Indeed, overexpression of telomerase is sufficient to counteract telomere attrition and to indefinitely extend the replicative lifespan of primary cells in culture in the absence of genomic instability, transforming them into cancerous cells ^20– 22^. However, high telomerase expression is normally restricted to early stages of embryonic development (i.e. the blastocyst stage in mice and humans) and to pluripotent embryonic stem cells ^23, 24^. Thus, adult mammalian tissues including adult stem cell compartments do not express sufficient amounts of telomerase to maintain telomere length throughout organismal lifespan. Consequently, telomere shortening occurs along with physiological aging in humans and mice and this process is proposed to underlie aging and age-associated diseases as well as organismal longevity ^25, 26^.

In addition to the core components TERT and TERC, the telomerase holoenzyme further consists of the accessory dyskerin complex composed of the proteins DKC1, NOP10, NHP2, and GAR1 ^27, 28^, which also play essential roles in telomere biology. Holoenzyme assembly is thought to occur in the Cajal bodies ^29^, and subsequently TCAB1 and TPP1 are required for proper trafficking of telomerase to telomeres. Moreover, the discovery of a long non-coding telomeric repeat-containing RNA, TERRA ^30, 31^, which has been proposed to regulate various aspects of telomere function, adds yet another level of complexity to telomere regulation ^32, 33^. Another crucial issue in telomere stability and maintenance is the replication of telomeric DNA, for which a myriad of proteins are required. Key factors in telomeric DNA replication are the CST complex (comprising the proteins CTC1, STN1, and TEN1) ^34, 35^, which facilitates telomere replication and simultaneously limits telomerase activity. WRN is a helicase with 3′ to 5′ exonuclease activity, which is also required for efficient telomere replication ^36^ as well as processing of the 3’ telomeric overhang ^37, 38^. The helicase BLM contributes to telomere stability by resolving late replication structures ^39^, whereas FEN1 and RTEL1 function in Okazaki fragment processing ^40^ and T-loop disassembly during replication ^41^, respectively. We recently published an in-depth review on the role of these proteins in telomere replication including the consequences for telomere maintenance if their function is impaired ^42^.

In this review, we will discuss the role of telomeres in the origin of age-associated diseases and organismal longevity, as well as the potential use of telomerase as a therapeutic target to delay aging and to prevent and treat age-related diseases.

## Telomeres as hallmarks of aging and longevity

Aging is a multifactorial process that results in a progressive functional decline at cellular, tissue, and organismal levels. During recent years, a number of molecular pathways have been identified as main molecular causes of aging, including telomere attrition, cellular senescence, genomic instability, stem cell exhaustion, mitochondrial dysfunction, and epigenetic alterations, among others ^26^. Interestingly, telomere attrition is considered a primary cause of aging, as it can trigger all the above-mentioned hallmarks of aging, although the degree to which it is a principal cause of aging is under active investigation ^26^. Critical telomere shortening elicits the induction of cellular senescence or the permanent inability of cells to further divide, which in turn has been proposed to be at the origin of different disease states ^17, 43^. In addition, telomere attrition in the stem cell compartments results in the exhaustion of their tissue- and self-renewal capacity, thus also leading to age-related pathologies ^44, 45^. Indeed, when this telomere exhaustion occurs prematurely owing to germline mutations in telomere maintenance genes (i.e. telomerase or shelterin genes), this triggers a premature loss of the renewal capacity of tissues leading to the so-called telomeropathies or telomere syndromes, including aplastic anemia and pulmonary fibrosis, among others ^46– 49^. Loss of DNA damage checkpoints can also allow the propagation of cells with short/damaged telomeres, thus leading to chromosome end-to-end fusions and genomic instability, as well as age-associated diseases like cancer ^50, 51^. A link between dysfunctional telomeres and mitochondrial compromise has been also proposed through transcriptional repression of the *PGC-1α* and *PGC-1β* genes by short telomeres, thus linking dysfunctional telomeres to mitochondrial aging ^52^. Finally, short telomeres can trigger epigenetic changes at telomeric as well as subtelomeric chromatin ^53^. In this regard, epigenetic regulation of telomeres has been described in processes that involve de-differentiation and loss of cellular identity such as during tumorigenesis ^54^, as well as during the induction of pluripotency ^55^. In particular, loss of heterochromatic marks at telomeres results in telomere elongation and increased telomere recombination ^53^.

Of note, in addition to the persistent DNA damage response elicited by critically short telomeres, it recently became evident that a large proportion of DNA damage in stress-induced senescence resides in telomeres. Importantly, this DNA damage is independent of telomere length and accumulates with aging in primates and mice, suggesting that stress-induced and telomere length-independent senescence may contribute to the aging process too ^56, 57^.

In addition to being considered a primary molecular cause of aging, telomere shortening with time has been proposed to be a biomarker of biological aging, with a potential prognostic value for many different age-associated diseases, including cardiovascular failure ^58– 64^. Interestingly, telomere length has also been proposed as a marker of longevity. A study longitudinally following telomere length throughout the lifespan of individual zebra finches demonstrated that telomere length at day 25 after birth is a strong predictor of individual lifespan in this species ^65^. In mice, a similar longitudinal follow up of telomere length throughout lifespan showed the rate of increase of short telomeres with time but not average telomere length or the rate of telomere shortening was predictive of individual lifespan ^66^. This study also showed for the first time that laboratory wild-type mice shortened telomeres at a pace that was 100-fold faster than humans, thus providing a potential explanation for shorter lifespans in mice (2–3 years) compared to humans, in spite of their long telomere length at birth (~50–150 kb in mice versus ~15–20 kb in humans) ^67, 68^. A similar scenario was found in dogs, where telomere shortening has been described to be 10-fold faster than in humans ^69^. These findings suggest that it is the ability of different species to maintain telomeres rather than average telomere length *per se* that may be determinant of species longevity. This idea is further supported by longitudinal studies in free-living birds. In particular, in Seychelles warblers, telomeres shorten throughout life and higher rates of telomere shortening predict mortality ^70^. Similarly, survival in jackdaws can be predicted by nestling telomere shortening but not by absolute telomere length ^71^.

Additional and independent evidence that the ability to maintain telomeres may determine mouse longevity came from the description of an age-specific metabolic signature predictive of chronological age in wild-type mice ^72^. In particular, when this signature was used to predict the age of either telomerase-deficient or *TERT*-overexpressing mice, it predicted older or younger ages than their chronological age, respectively, in agreement with shorter telomeres and shorter lifespan in the telomerase-deficient mice, and longer telomeres and extended lifespan in the *TERT*-overexpressing mice ^72^, thus suggesting that telomere length is a determinant of aging in wild-type mice.

In humans, a large number of cross sectional epidemiological studies confirmed telomere shortening with aging in humans ^16, 73^. Recently published data from the GERA cohort (Genetic Epidemiology Research on Adult Health and Aging), which comprises more than 100,000 individuals, further confirmed this correlation and also showed that telomere length correlates positively with survival in subjects older than 75, i.e. longer telomeres provide more years of life ^74^. This is in agreement with a previous report showing that telomere length positively correlates with better median survival in individuals who are 60 years of age or older ^75^. However, contradictory reports exist which do not support the correlation between average telomere length and the prediction of remaining years of life in the old and oldest ^76, 77^. In this regard, lessons from other species (mice, birds) show the importance of determining not only average telomere length but also longitudinal changes in telomere length as well as changes in the abundance of short telomeres. Thus, future epidemiological studies should take individual telomeres and their change over time into account (i.e. the rate of increase of the fraction of short telomeres). In this regard, methods that can quantify the presence of short telomeres, like the high-throughput quantitative telomere fluorescence *in situ* hybridization (FISH) technique ^58^ or single telomere length analysis (STELA) ^78^ will be important for establishing telomere shortening as a biomarker of human aging.

## Intrinsic and environmental instigators of telomere length

As mentioned above, there are differences in the pace of telomere shortening across species, which indeed may contribute to explaining their different longevities, at least in part. The average telomere shortening in human blood cells occurs at a rate of 31–72 base pairs per year ^79, 80^ while mouse telomeres shorten around a hundred times faster than that ^66^. This indicates that, in addition to the intrinsic end replication problem, there are other factors contributing to telomere attrition. In particular, oxidative damage may severely impact on telomere length. Cells exposed to oxidative stress conditions (e.g. H _2_O _2_, chronic hyperoxia) display accelerated telomere shortening and reduced replicative lifespans, whereas antioxidant treatment has the opposite effect ^81^. In humans, the choice of lifestyle can influence telomere shortening. As an example, smoking, an unhealthy diet (e.g. high cholesterol, alcohol intake), or obesity might lead to telomere shortening by provoking tissue inflammation and oxidative stress ^82– 87^. Moreover, accelerated telomere shortening in leukocytes has been associated with psychological stress. In particular, patients with depression disorders have shorter telomeres compared to healthy individuals ^88^, and this telomere erosion is found in all lymphocyte subpopulations of the adaptive immune system ^89^. Stress provoked by physical abuse of children has been also associated with telomere shortening ^90^. Furthermore, there is a wealth of studies investigating telomere length in major depressive disorder (MDD), a severe illness which shows signs of premature aging ^60, 91, 92^. In particular, it has been described that telomere length in MDD subjects corresponds to a 10-year increase in biological age ^93^ compared to healthy subjects. In line with this, increased abundance of short telomeres in patients with bipolar II disorder has also been described to correspond to a 13-year older biological age, again in agreement with increased risk for developing different diseases in these patients ^94^. Interestingly, shorter telomeres are also associated with cognitive impairment in the elderly ^58^.

In contrast to the detrimental factors causing accelerated telomere shortening, certain life habits (e.g. a diet rich in omega-3 fatty acids) ^81, 95^, as well as physical activity, exercise, and fitness, have been proposed to reduce telomere erosion and thus slow down the pace of aging ^96– 98^.

In addition to these various intrinsic and environmental factors, telomere length is also dictated by a genetic component. Earlier twin and family studies and a recent meta-analysis comprising nearly 20,000 subjects demonstrate that telomere length is highly heritable ^79, 99– 101^. Whether the inheritance of telomere length correlates more strongly with paternal or maternal telomere length, however, is still debated ^102^. Interestingly, in another twin study Christensen and colleagues reported that the perceived age in twins older than 70 years of age is a robust biomarker of aging which strongly correlates with telomere length. Moreover, within twin pairs, the twin with greater telomere length tends to look younger and live longer ^103^.

## Genetic models to understand the causal role of telomeres in disease and longevity

 Firm experimental demonstration that critical telomere shortening is causative of aging was first achieved by generating mice deficient for telomerase. Mice deficient for *TERC* have progressively shorter telomeres over generations, leading to chromosome instability, developmental defects, premature aging phenotypes, and ultimately mouse infertility and premature death ^104– 106^. These mice show a decreased median and maximum lifespan already at the first generation ^107^, and this decreased longevity and associated aging pathologies are anticipated with each mouse generation, thus demonstrating that telomere length in mice is causal of aging and longevity. Importantly, restoration of *TERC* expression in mice with inherited critically short telomeres is sufficient to prevent the phenotypes associated with short telomeres in these mice, including aplastic anemia, intestinal atrophy, and infertility, among others ^108, 109^. In agreement with these pioneer studies, genetic ablation of *TERT* was shown to have similar consequences on organismal aging and lifespan ^110, 111^. Furthermore, *TERT* reconstitution in late generation *TERT-*deficient mice also led to telomere elongation, lower DNA damage load, and reversal of degenerative phenotypes in these mice ^112^. In line with these findings, lack of telomerase in lower vertebrates such as the zebrafish also causes premature aging which can be rescued by either telomerase restoration or inhibition of p53, which signals telomere damage ^113^. Together, these findings demonstrate that short telomeres are causative of aging and that premature aging specifically induced by telomerase deficiency and short telomeres can be rescued by telomerase re-expression.

 In line with mouse studies, a number of human syndromes were later described to be caused by germ line mutations in telomerase and shelterin genes, the so-called telomere syndromes ^47^. As in the telomerase-deficient mouse model, the diseases associated with telomerase mutations are anticipated with increasing generations and involve a loss of the ability of tissues to regenerate, resulting in skin abnormalities, aplastic anemia, or pulmonary fibrosis ^46, 47^. These analogies between humans and mice highlight that telomere length as a genetic determinant of disease and longevity is a molecular mechanism conserved in these species.

However, definitive genetic demonstration that telomere length is also causative of physiological aging in normal individuals first came from telomerase overexpression studies in mice. In particular, mice with increased transgenic telomerase expression throughout their lifespans were able to maintain longer telomeres with aging, showed decreased molecular (i.e. lower DNA damage) and physiological biomarkers of aging, showed a delayed appearance of age-related pathologies (osteoporosis, metabolic decline, etc.), and showed a significant increase in organismal longevity. In particular, transgenic *TERT* overexpression in mice engineered to be cancer resistant resulted in decreased incidence of aging-related pathologies and a striking 40% extension of median survival compared to wild-type mice ^114^. This study demonstrated for the first time in any organism the anti-aging activity of telomerase. Importantly, these findings led to the idea that potential therapeutic strategies based on transiently increased telomerase expression could also delay age-associated pathologies and increase longevity. This was first achieved by delivering *TERT* using non-integrative gene therapy vectors (adeno-associated vectors [AAVs]) into middle-aged and old mice, which resulted in transiently increased *TERT* expression in the majority of mouse tissues. Importantly, a single treatment with these vectors resulted in elongated telomeres in a range of organs, delayed age-associated pathologies, and significantly extended median and maximal lifespan in both age groups ^115^. Moreover, these mice did not show increased cancer; instead, as seen in other age-related conditions, cancer was also delayed ^115^. Thus telomere-based gene therapies using non-integrative vectors may represent a new therapeutic strategy to transiently activate *TERT* for the prevention or treatment of many different age-related pathologies (see below).

## Telomeres and Telomerase as therapeutic targets

A substantial number of companies are now aiming to harness the knowledge that has been generated, unveiling the molecular mechanisms of aging in order to develop a new class of drugs to prevent and treat the major age-related diseases ^116^. In this regard, telomerase overexpression studies in mice have been proof of principle that just modifying a single hallmark of aging, i.e. telomere shortening, this was sufficient to delay not one but many different age-associated pathologies in mice, including cognitive decline ^114, 115^. Indeed, the use of telomerase activation in delaying aging-associated conditions has spurred the interest of commercial enterprises. For instance, the low-potency telomerase activator TA-65 (a bio-active compound isolated from the herb *Astragalus membranaceus*) has been shown to lead to a mild increase in telomere length in mice ^117^, zebra finches ^118^, and humans ^119^, and to improve several aging-related parameters in mice and humans ^117, 119^, although no increase in longevity has been reported in longitudinal mouse studies ^117^. On the other hand, other natural compounds like sex hormones have been found to activate *TERT* at the transcriptional level ^120– 122^. In this regard, androgen therapy has been applied as a first-line treatment in aplastic anemia for decades with mixed success and without a clear understanding of the mechanism that underlies remission in some patients but not in others ^123, 124^. A recent study in mice which develop full-blown aplastic anemia provoked by short telomeres showed that androgen therapy rescues telomere attrition and subsequent death from aplastic anemia ^122^, indicating that telomerase activation may indeed be a treatment option for diseases associated with flawed telomere maintenance (i.e. telomeropathies or telomere syndromes). However, potential off-target effects of compounds that activate *TERT* at a transcriptional level should be a concern. In particular, TA-65 has been shown to activate *TERT* through activation of mitogenic pathways that lead to the activation of the oncogene c-myc ^117, 125^ and thus may drive cancer. Interestingly, such off-target effects may be circumvented through direct delivery of *TERT*, such as by means of systemic gene therapy using non-integrative AAV vectors, which showed a significant delay of age-related pathologies in mice and increased longevity ^115^. A recent study using fibroblasts *in vitro* also proposed delivery of the *TERT* mRNA as a way to activate telomerase ^126^. However, it should be mentioned that strategies for telomerase activation, indirect or direct, have raised safety concerns due to the close correlation of most cancers and constitutive reactivation of endogenous telomerase. This highlights that, in addition to proof-of-concept studies in mice, the development of safe strategies for transient and controllable telomerase activation in humans should be a future goal.

In this regard, *TERT* gene therapy with AAVs is particularly attractive for *TERT* activation, since the non-integrative and replication-incompetent properties of AAVs allow for cell division-associated telomere elongation and subsequent loss of *TERT* expression as cells divide, thus restricting *TERT* expression to a few cell divisions. Thus, this strategy assures a transient and relatively genome-safe *TERT* activation. In contrast, the use of *TERT* mRNA currently lacks appropriate systems for *in vivo* delivery, and thus its use may be restricted to *ex vivo* applications.

It is likely that the first clinical use of a *TERT*-based therapy, such as the *TERT* gene therapy approach developed by us, will be for the treatment of the human telomere syndromes, including aplastic anemia and pulmonary fibrosis. However, this requires the development of appropriate preclinical models and the subsequent clinical trials in humans. In this regard, we have recently generated two mouse models which recapitulate the clinical features of aplastic anemia ^127^ and pulmonary fibrosis ^128^. The disease in both models is provoked by short and dysfunctional telomeres and thus these models provide a platform for further testing of *TERT*-based treatment strategies for the telomere syndromes.

Given that physiological aging is provoked, at least in part, by telomere shortening, a *TERT* gene therapy may be used not only for the prevention and treatment of telomere syndromes but also for the treatment of multiple age-related diseases. In this regard, short telomeres have been extensively associated with a higher risk for cardiovascular disease ^64, 129, 130^. In support of a potential use of *TERT* activation in the treatment of age-related diseases, we demonstrated that *TERT* gene therapy can efficiently rescue mouse survival and heart scarring in a preclinical mouse model for heart failure upon induction of acute myocardial infarction ^131^.

**Figure 1.**
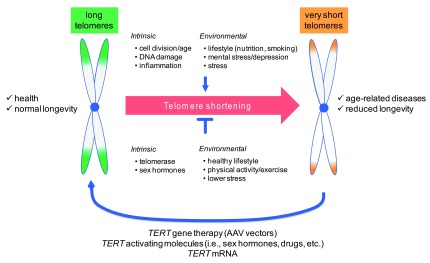
Telomeres in aging and disease. Telomere shortening is a life-long process that is influenced by a number of intrinsic and environmental factors that either accelerate or slow down natural telomere attrition, which causes aging and the emergence of age-related diseases. The identification of telomere shortening as a driver of molecular aging has triggered the development of telomerase-based strategies to (re)elongate telomeres and thus to delay aging and associated disease. Abbreviations: AAV, adeno-associated vectors; TERT, telomerase reverse transcriptase.

Collectively, experiments in cell and animal models provide proof of concept for the feasibility of telomerase activation approaches to counteract telomere shortening and its consequences ( Figure 1). In particular, the successful use of telomerase gene therapy in animal models of aging and short telomere-related diseases paves the way for the development of therapeutic telomerase treatments in human aging and associated disease.
