# Supplementary material for: Telomeres and telomerase as therapeutic targets to prevent and treat age-related diseases
Source: F1000Res. 2016 Jan 20;5:F1000 Faculty Rev-89. [Version 1] doi: 10.12688/f1000research.7020.1 (PMC4815611; doi:10.12688/f1000research.7020.1)
Supplement: Peer review history

# Peer review: Referee response for version 1

**DOI:** 10.5256/f1000research.7558.r12015

**Published:** 2016-01-21

**Authors:** F. Bradley Johnson

**Affiliations:** 1 Department of Pathology and Laboratory Medicine, University of Pennsylvania, Philadelphia, PA, USA

Until it is demonstrated that enhanced telomere maintenance delays or reverses age-related pathologies in humans, it will not be know for certain to what extent telomere dysfunction contributes to natural human aging.  Nonetheless, the current evidence, as reviewed by Bar and Blasco, does suggest that targeting telomeres will prove to be helpful for the amelioration of age-related diseases.

I have read this submission. I believe that I have an appropriate level of expertise to confirm that it is of an acceptable scientific standard.

# Peer review: Referee response for version 1

**DOI:** 10.5256/f1000research.7558.r12014

**Published:** 2016-01-21

**Authors:** Lucio Comai

**Affiliations:** 1 Department of Molecular Microbiology and Immunology, University of Southern California, Los Angeles, CA, USA

I have read this submission. I believe that I have an appropriate level of expertise to confirm that it is of an acceptable scientific standard.

# Peer review: Referee response for version 1

**DOI:** 10.5256/f1000research.7558.r12013

**Published:** 2016-01-21

**Authors:** Fabrizio d'Adda di Fagagna

**Affiliations:** 1 IFOM - FIRC Institute of Molecular Oncology Foundation, Milan, Italy

I have read this submission. I believe that I have an appropriate level of expertise to confirm that it is of an acceptable scientific standard.

# Peer review: Referee response for version 1

**DOI:** 10.5256/f1000research.7558.r12012

**Published:** 2016-01-21

**Authors:** Jerry Shay

**Affiliations:** 1 Cell Biology, University of Texas Southwestern Medical Center, Dallas, TX, USA

I have read this submission. I believe that I have an appropriate level of expertise to confirm that it is of an acceptable scientific standard.
